# Supplementary material for: Elevated Flotillin-1 in Saliva and Salivary Glands: A Novel Non-Invasive Biomarker in an Alzheimer’s Disease Mouse Model
Source: Diagnostics (Basel). 2025 Dec 24;16(1):61. doi: 10.3390/diagnostics16010061 (PMC12785311; doi:10.3390/diagnostics16010061)
Supplement: Supplementary file 1 [file diagnostics-16-00061-s001.zip › diagnostics-4003115-supplementary.pdf]

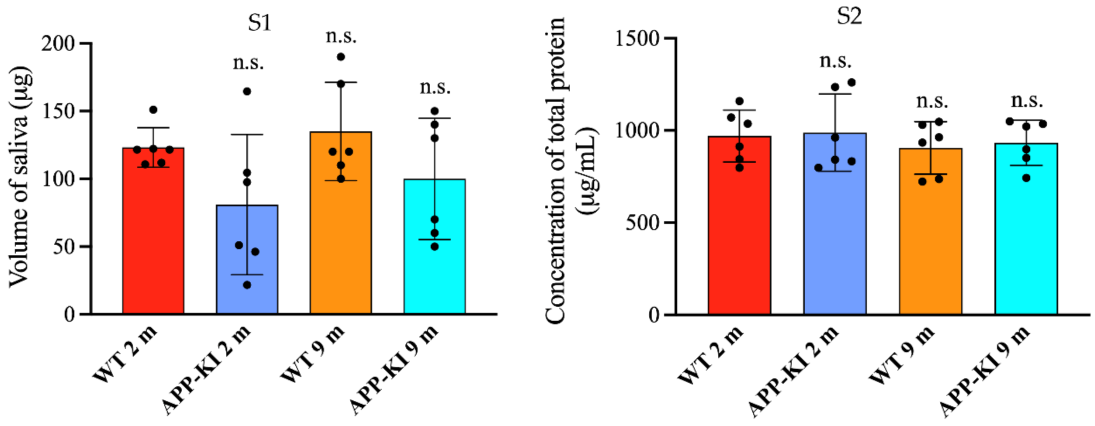

Figure S1 is Salivary secretion in amyloid precursor protein knock-in (APP-KI) and wild-type (WT) mice. Figure S2 is total protein concentration in the saliva of amyloid precursor protein knock-in (APP-KI) and wild-type (WT) mice. Salivary secretion and total salivary protein concentrations were compared between APP-KI and WT mice at 2 and 9 months of age. Data were analyzed using the Kruskal–Wallis test, followed by the Steel–Dwass post-hoc test for multiple comparisons. Data are presented as mean ± SD. n = 6 per group. No significant differences were observed among the groups ($p > 0.05$).
